# A bio-pen for direct writing of single molecules on user-functionalized surfaces†

**DOI:** 10.1039/c9na00379g

**Published:** 2019-10-31

**Authors:** Xiao Hu, Cerasela Zoica Dinu

**Affiliations:** Department of Chemical and Biomedical Engineering, West Virginia University, Benjamin M. Statler College of Engineering and Mineral Resources PO Box 6102 Morgantown WV 26506 USA cerasela-zoica.dinu@mail.wvu.edu +1 304 293 4139 +1 304 293 9338

## Abstract

Advancing ultrahigh resolution (below 10 nm) direct writing technologies could lead to impacts in areas as diverse as disease detection, genetic analysis and nanomanufacturing. Current methods based on electron-beams and photo- or dip-pen nanolithography are laborious and lack flexibility when aiming to create single molecule patterns for application specific integration. We hypothesize that a novel strategy could be developed to allow for writing of parallel and yet individually addressable patterns of single molecules on user-controlled surfaces. The strategy is based on using *in vitro* self-recognition of tubulin protein to assemble rigid protofilaments of microtubules, with one such microtubule to be subsequently used as a “bio-pen” capable of writing “inks” of single kinesin molecules in user-defined environments. Our results show that single kinesin inks could be written under the energy of adenosine triphosphate hydrolysis and observed by both atomic force and optical microscopy. Upon extending ink functionalities, the integration of soft and hard materials for nanostructure assembly and complex single molecule pattern formation is envisioned.

## Abstract 2

Single kinesin molecules printed onto a user-functionalized substrate using biological pens.

## Graphical abstract

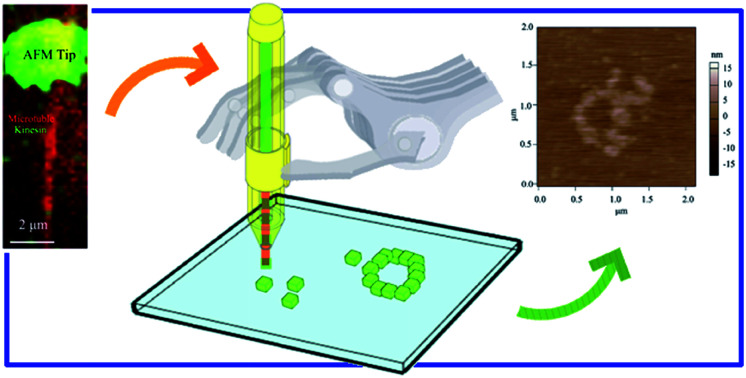

## Introduction

Single molecule or component direct writing could impact areas as diverse as nanomaterial and nanostructure assemblies, disease detection and genetic analysis. Current methods for advancing nanomaterial/nanostructure formation are based on electron-beam lithography,^1^ photolithography,^2^ polymer pen lithography,^3^ or micro-contact printing^4^ and are used to deposit or assembly dots, lines and arrays. Using electron-beam lithography for instance led to custom nanostructures' formation at a resolution of about 60 nm for the facile fabrication of nanosensors.^5^ Hierarchical super-hydrophobic well-ordered secondary nanostructured surfaces were successfully produced by dual-scale electron-beam lithography for self-cleaning applications.^6^ In disease detection, UV nanoprinting led to the formation of large area arrays of goat anti-human kappa chains for fast, low-cost screening of leukemic cancer markers.^7^ Similarly, rapid-disease screening assays for advancing genetic analysis immunoarrays^8^ were developed using microcontact printing; the same technique was also applied for the formation of DNA arrays^9^ for ultrasensitive and simultaneous detection of the cancer biomarker prostate specific antigen (PSA) and interleukin-6 (IL-6) proteins in serum, all at sub-pg mL^−1^ levels. However, the above printing technologies do not work routinely in the sub 50 nm regime, especially when features are to be made from mixed hard and soft materials.^8^ Furthermore, laborious and difficult steps limit the techniques' flexibility when aiming to generate parallel and yet individual patterns of multicomponent nanostructures to allow for increased integration and flexibility. Finally, the light or heat sensitivity of the proteins and oligonucleotides used in genetic and disease testing platforms needs integration of complex, additional and separate steps to be used for fabrication, with many such steps known to reduce both the activity and functionality of the prints.^10^

To increase flexibility for manufacturing and implementation, dip-pen nanolithography (DPN) and single-molecule cut-and-paste bottom up assembly were introduced as viable alternatives. The high resolution, registration and direct-writing ability in both ambient and inert environments allowed the formation of specific structures such as dots, lines and circles while eliminating etching and avoiding cross-contamination^11^ normally associated with the methods listed above. As such, the gentle writing ability was shown to lead to the formation of patterns of biologically active proteins used for studying hierarchical assembly processes of systems,^12^ while DNA arrays^13,14^ were used for addressing bio-sensing-related sample sensitivity. However, even though increased versatility was achieved, such methods continue to rely on non-specific forces for transferring an “ink” onto a “paper” which could lead to propagation of contaminants.^15^ Furthermore, DPN lacks the ability to transfer individual molecules in a one-by-one fashion onto nanometer-sized spaces and is only capable of achieving about 20 nm resolution^12^ while the cut and paste method is highly restricted by its customization.^14^ The next generation of direct writing technologies should not only allow for individual separation of single molecules, but also for parallel and yet individual sustainability, and attainability of the molecule or written components' diversity, all while increasing randomness and complexity of the deposited materials under ambient temperature and pressure conditions respectively.

We propose to design the next generation of biological tools capable of creating flexible single molecule patterns that have a high degree of individual, yet parallel characteristics and could be used for site-specific transformations and integration into protein-driven nanomanufacturing strategies. Our strategy is based on using a user-friendly approach in which a microtubule cytoskeletal filament serves as an affixed “biological pen” capable of being manipulated *in vitro* to thus provide the route for writing single molecules of kinesin 1 motor proteins, a.k.a. kinesin “inks”. In the cell, microtubules give structural integrity while serving as regular and uniform tracks for the transport of vesicles or organelles; microtubules are growing with their plus end (fast growing end) towards the cell periphery while the slow end (or minus end) is oriented towards the cell nucleus. Kinesin uses a microtubule track *in vivo* to progress to specific locations with processive and coordinated 8 nm steps and *in vitro* with speeds below 1 μm s^−1^, all under the transformation of the chemical cycle of adenosine triphosphate (ATP) into mechanical work.^16^ Our strategy demonstrates the ability to write individually addressable kinesin patterns, all with nanometer resolution, on user-engineered surfaces. Considering the genetic capability for kinesin functionalization, it is envisioned that different kinesin-tagged inks and thus complex nanostructure writing capabilities could be introduced, all to allow the formation of single molecule patterns with high feasibility and versatility for advancing areas as diverse as nanomanufacturing and disease detection.

## Materials and methods

### Expression of a fluorescent labeled kinesin molecular motor

The plasmid pPF_dmKHC-EGFP encoding for the dmKHC-EGFP protein (molecular weight 91.7 kDa) consisting of the *Drosophila melanogaster* kinesin delta tail (dmKHC) linked to the C-terminal end of the EGFP (enhanced green fluorescent protein) his-tagged on the protein's C-terminus was obtained at West Virginia University. The coding sequence was copied by polymerase chain reaction (PCR) from the pPK124 plasmid, a kind gift of Prof. Jonathan Howard, Yale University. Briefly, a set of primers of known sequences (CAAAGGAGATATACATATGAGCGCAGAACGAGAAATTCC and CTCGCCCTTGCTCAC.GCTCCCACGCGGAACAAG respectively) were used; the pPF_EGFP plasmid was a pTriEx-4 plasmid (Novagen, MA, USA) carrying the coding sequence for a C-terminal his-tagged EGFP.

The pPF_EGFP plasmid was first linearized by treatment with the *NcoI* endonuclease (New England Biolabs, USA). Both the linearized pPF_EGFP plasmid and the pPK124 PCR amplicon were then gel purified and assembled using a Gibson assembly kit (New England Biolabs, USA) as per the manufacturer's protocol. The assembled DNA sequences were subsequently introduced into *E. coli* strain Stbl4 (Invitrogen, Fisher Scientific, USA); clones were screened by endonuclease treatment and small-scale protein expression followed by standard lab purification was applied.

The pPF_dmKHC-EGFP plasmid was transformed into the *E. coli* strain BL21(DE3) pLysS (Stratagene, Agilent Technologies, USA). Protein expression was induced with 1.0 mM Isopropyl β-d-1-thiogalactopyranoside (IPTG) at 16 °C for 18 h. The cell pellet was subsequently resuspended in ice-cold lysis buffer containing 50 mM tris(hydroxymethyl)aminomethane hydrochloride (Tris/HCl), pH 8.0, 50 mM sodium chloride (NaCl), 2 mM magnesium chloride (MgCl_2_), 0.1 mM adenosine 5′-triphosphate (ATP), 2% Triton X-100, 10 mM β-mercaptoethanol, 0.4 mg mL^−1^ lysozyme, ethylenediaminetetraacetic acid (EDTA)-free protease inhibitor cocktail (reagents from http://Biotool.com, USA), 1 mM phenylmethylsulfonyl fluoride (PMSF), 8% trehalose, and 10 U mL^−1^ of Pierce Universal Nuclease (Fisher Scientific, USA), in a volume of 10 mL of lysis buffer per 1 g of cell pellet. Any cellular debris was removed by centrifugation (performed at 20 000 g, 4 °C, for 10 min), and the clear lysate was supplemented with 4 M NaCl for a final concentration of 0.5 M.

The expressed protein was purified using two Bio-Scale Mini IMAC cartridges (Bio-Rad Laboratories Inc., USA) in series installed in a BioLogic DuoFlow chromatography system (Bio-Rad Laboratories Inc., CA, USA) by running two buffers, *i.e.*, the wash buffer (50 mM sodium phosphate (Na_3_PO_4_), 0.3 M NaCl, 1 mM MgCl_2_, 10 μM ATP, 5 mM β-mercaptoethanol, 10% glycerol, pH 8.0) and the elution buffer (wash buffer with 0.5 M imidazole) respectively. The protein was then eluted with an imidazole gradient and concentrated to 1 mL volume. A second chromatographic step was applied for further purification; specifically, a Superdex 200 10/300 GL gel-filtration column (GE Healthcare Life Sciences, USA) equilibrated with the storage buffer (100 mM imidazole, 300 mM NaCl, 1 mM MgCl_2_, 10 μM ATP, 1.0 mM 1,4-dithiothreitol (DTT), 10% sucrose, pH 7.0) was used. Finally, the protein concentration was estimated using the Coomassie protein assay and bovine gamma globulin standard (Fisher Scientific, USA), with the purification process monitored by classic sodium dodecyl sulfate polyacrylamide gel electrophoresis (SDS-PAGE).

### *In vitro* synthesis of microtubules

Microtubules were synthesized from free tubulin suspended in a microtubule polymerization solution. Briefly, the polymerization solution was obtained by vortexing 5 μL 100 mM MgCl_2_, with 6 μL dimethyl sulfoxide (DMSO, 99.7%, Fisher Scientific, USA), 5 μL 25 mM guanosine-5′-triphosphate (GTP, Sigma, USA) and 9 μL BRB80 buffer (formed from a mixture of 80 mM piperazine-*N*,*N*′-bis(2-ethanesulfonic acid) buffer, 1 mM MgCl_2_ and 1 mM ethylene glycol tetraacetic acid (EGTA), pH 6.8; all reagents were purchased from Fisher Scientific, USA). To initiate microtubule polymerization, 2.5 μL polymerization solution was mixed with 10 μL of 4 mg mL^−1^ biotin and rhodamine labeled tubulin (Cytoskeleton Inc, USA) and the mixture was incubated at 37 °C for 30 min. To stabilize the resulting microtubules, the solution was dispersed in 1 mL BRB80 buffer containing 10 μM paclitaxel (Fisher Scientific, USA). The stabilized microtubules were kept at room temperature for future experimental use.

### Microtubule inking with kinesin molecules

Kinesins' ability to bind to the lab-synthesized microtubules was evaluated using a non-hydrolyzable form of ATP. Briefly, 8 μL of 10 μg mL^−1^ kinesin expressed as previously described was mixed with 2 μL 20 mM adenylyl-imidodiphosphate (AMP-PNP, Sigma, USA) and incubated for 1 h at 4 °C. The mixture was subsequently mixed with 20 μL microtubules (prepared as previously described) and 10 μL of BRB80 buffer containing 10 μM paclitaxel; the resulting solution was incubated for 30 min at room temperature. Upon incubation, the unbound kinesin was separated in the supernatant by using an Allegra 64R centrifuge (Beckman Coulter, USA) and 30 000 rpm spinning for 10 min. The supernatant was evaluated using fluorescence microscopy (Nikon, USA). The resulting pellet was dissolved in 40 μL BRB80 containing 10 μM paclitaxel and used immediately.

### Glass substrate functionalization

Glass substrates were coated with anti-kinesin antibodies using a covalent binding strategy. For this, glass slides (*d* = 25 mm, Corning, USA) were first ultrasonicated in DI water, 99% ethanol (90%, Fisher Scientific, USA), and again in DI water, with 30 min for each of the sonication windows. Second, the slides were dried under vacuum for 1 day and then exposed to UV light for 30 min. Third, the glass slides were treated with piranha solution (mixture of 96.4% sulfuric acid, H_2_SO_4_, and 30% hydrogen peroxide, H_2_O_2_, Fisher Scientific, USA) in a 3 : 1 volume ratio at 120 °C for 10 min. Such cleaned glass slides were subsequently washed with DI water and dried under vacuum for an additional day. Upon drying, the slides were immersed in 1 mL of 5% 3-aminopropyltriethoxysilane (APTES; Fisher Scientific, USA) in toluene (99.5%, Fisher Scientific, USA) and incubated at room temperature for 1 h. Upon lapse of time, the slides were washed thoroughly with DI water, toluene and DI water and subsequently immersed in 1 mL 5% glutaraldehyde (Acros Organics, USA) in 0.2 M pH 9.0 Tris-buffered saline (TBS, made from tris and hydrochloric acid, reagents purchased from Fisher Scientific, USA) at room temperature, for 1 h, with shaking at 200 rpm. Finally, the slides were extensively rinsed with TBS, activated for 15 min in 1 mL 160 mM 1-ethyl-3-(3-dimethylaminopropyl)carbodiimide (EDC, Acros Organics, USA) and 80 mM *N*-hydroxysuccinimide (NHS, Pierce, USA) in 2-(*N*-morpholino)ethanesulfonic acid buffer (MES, pH 4.7, Fisher Scientific, USA) and finally incubated in 1 mL of 1 mg mL^−1^ anti-kinesin antibody (Antibodies Online, USA) in BRB80 at room temperature and 200 rpm for 3 h respectively. Upon incubation, the functionalized glass slides were rinsed thoroughly with BRB80 to remove loosely bound antibodies.

### Functionalization of an atomic force microscope tip

A TR-400PB tip (Asylum Research, USA) was functionalized with kinesin-inked microtubules. For this, the tip was first cleaned by immersion in 1 mL of deionized (DI) water for 10 min, second by immersion in acetone (99.5%, Fisher Scientific, USA) for another 10 min, and third by immersion in DI water for an additional 10 min. Subsequently, the tip was dried under air, exposed to UV light for 30 min, rinsed with 100 mM pH 7.0 phosphate buffered saline (PBS, made from mono-potassium phosphate (KH_2_PO_4_), dipotassium hydrogen phosphate (K_2_HPO_4_) and NaCl, all reagents were purchased from Fisher Scientific, USA) and incubated in a solution of 50 mg mL^−1^ anti-tubulin antibody (Sigma, USA) for 3 h at 4 °C. Upon incubation, the tip was rinsed with BRB80 to remove loosely bound antibodies. Finally, 40 μL kinesin-inked microtubule solution was dropped onto the tip surface and incubated for 10 min at room temperature; upon incubation, the tip was rinsed with BRB80 buffer containing 10 μM paclitaxel (ESI Scheme S1†). The functionalized tip was used immediately upon preparation (see below).

### Kinesin writing onto a functionalized glass substrate

The inked tip was loaded onto the AFM head of a MFP-3D Bio (Asylum Research, USA) and engaged in close proximity of the anti-kinesin antibody functionalized substrate in the presence of 100 μL gliding solution (BRB80 buffer containing 1 mM MgATP and 20 mM d-glucose; both reagents were from Fisher Scientific, USA), 0.02 mg mL^−1^ glucose oxidase, 0.8 mg mL^−1^ catalase (both reagents were from Sigma, USA) and 0.5% β-mercaptoethanol (Fisher Scientific, USA). The set point as well as the deflection and trigger points were set at −0.5 V, −2.5 V and 3 nN respectively. The AFM head/tip was lowered manually until the *Z* voltage decreased to about 70 V. Subsequently the tip was engaged in the gliding solution for 1 min and 5 min to realize the writing process in contact mode (ESI Scheme S1†).

Kinesin writing onto anti-kinesin functionalized glass slides was evaluated using an ATP-based assay and observed using both fluorescence microscopy and AFM. For this, 100 μL gliding solution (BRB80 buffer containing 1 mM MgATP and 20 mM d-glucose; both reagents were from Fisher Scientific, USA), 0.02 mg mL^−1^ glucose oxidase, 0.8 mg mL^−1^ catalase (both reagents from Sigma, USA) and 0.5% β-mercaptoethanol (Fisher Scientific, USA) were added to the tip functionalized with the kinesin-inked microtubule and incubated for 5 min at room temperature. The writing process was observed using a fluorescence microscope and a 100× objective (NA = 1.4) under DAPI and GFP filters and under an exposure time of 8.3 s.

### Nanoscopic morphological analyses

Morphological analyses of microtubules, kinesin, antibody-functionalized surfaces and surfaces containing the printed kinesins were performed using a MFP-3D Bio in contact mode. For these, the cantilever's spring constant was calibrated using the thermal noise method and the scan rate of the tip was fixed at 0.5 Hz. At least 6 experiments for each one of the samples were performed.^17,18^

For microtubules, mica sheets washed with DI water, ethanol and again with DI water, and subsequently dried overnight under vacuum and at room temperature, were used as substrates. Mica was functionalized with 20 μL APTES through incubation at room temperature for 15 min. Upon incubation, mica was washed with toluene and DI water; any remaining solution was removed under vacuum. For the physical characteristics of the microtubules, 20 μL of the sample with 0.5% glutaraldehyde was dropped onto the APTES functionalized mica and incubated for 1 h at room temperature. After incubation, the surface was washed with 40 μL BRB80 containing 10 μM paclitaxel at least 3 times and subsequently imaged.

Contact mode AFM in the respective buffer of each sample was performed to evaluate changes in surface roughness.^17–21^ The obtained images were flattened to remove curvature and slope effects. The root mean square average of height deviation (as taken from the mean image data plane) and the arithmetic average of absolute values of the surface height deviations (*i.e.*, *R*_q_ and *R*_a_) were calculated from the obtained flattened images. Analyses were performed on the clean glass surface, APTES functionalized glass slide surface, APTES-glutaraldehyde-anti-kinesin antibody functionalized surface and APTES-glutaraldehyde-anti-kinesin antibody-kinesin functionalized surfaces, with the chosen areas for evaluation being 5 μm × 5 μm. The roughness of the APTES-glutaraldehyde-anti-kinesin antibody-kinesin functionalized surface was also evaluated on 1 μm × 1 μm areas. Finally, for evaluation of the printed kinesin, the AFM tip was removed from the buffer after the printing process (1 min and 5 min respectively) washed thoroughly with BRB80 buffer containing 10 μM paclitaxel and subsequently imaged under an AFM as described above.

## Results and discussion

Nanoscale printing or direct writing has revolutionized applications ranging from analysis of biochemical mixtures, to medicine and molecular-scale electronics. However, in such applications, there are challenges associated with the high-throughput nature of the printed patterns or arrays of patterns, as well as with the limited flexibility for parallelization to allow for single molecule placement under an ultrahigh resolution capability (below 10 nm).^22^

We hypothesize that a novel strategy could be developed to demonstrate direct writing of parallel and yet individually addressable patterns of single molecules on user-controlled surfaces. The strategy is based on using rigid filaments of cellular microtubules as pens of nm diameter to write inks of single kinesin molecules, in user-defined environments.

**Fig. 1:**
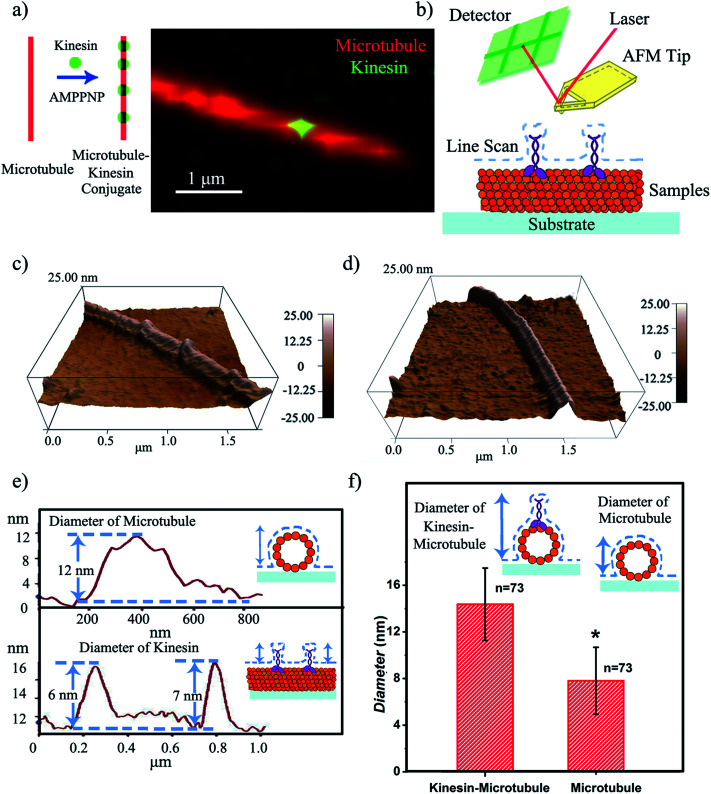
(a) Proposed kinesin-inked microtubule pen; optical microscopy allows microtubule identification in red and kinesin in green. (b) Schematic of the contact mode AFM analysis of a kinesin-inked microtubule pen. (c) Representative morphology of a kinesin-inked microtubule pen. The beads represent kinesin molecules bound to the immobilized microtubule, all under the chemical energy of adenylyl-imidodiphosphate (AMPPNP). (d) Representative morphology of a surface immobilized microtubule used as control. (e) Height profile analysis helps evaluate the distributions of kinesin inks on the microtubule pen. Upper image: readings are taken across the sample to evaluate the changes in the microtubule height resulting upon the binding of individual kinesin. Lower image: readings follow the sample's profile and help identify the change in the microtubule's local diameter as a change in its height profile when a bound kinesin is encountered. (f) Average diameters of the kinesin-inked microtubule pens and control microtubules respectively. Student's *T*-test considered a significance level of *p < 0.05.

To define the characteristics of the hypothesized writing strategy, we first demonstrated the feasibility of creating the microtubule pen (*i.e.*, bio-pen). Specifically, we used rhodamine labeled tubulin as the precursor for polymerizing microtubules *in vitro* under the chemical energy of guanosine triphosphate (GTP). The overall bio-pen (or microtubule pen) length was evaluated using optical microscopy (ESI,† Part 2). The resulting bio-pen was “inked” with lab-expressed green fluorescent kinesin (Fig. 1a). Kinesin's ability to ink the microtubule pen was evaluated under the non-hydrolyzable form of adenosine-5-triphosphate (ATP), *i.e.* adenylyl-imidodiphosphate (AMPPNP), known to be acting as a kinesin-microtubule binding stabilizer. Previous studies showed that the kinesin-AMPPNP complex recognizes and binds to the microtubule stably with a submicromolar *k*_d_ and a *k*_off_ of ∼0.0025 s^−1^.^23^

Contact mode atomic force microscopy (AFM) was used to confirm kinesin's ability to recognize the microtubule pen and evaluate its overall inking efficiency. For this, the microtubule inked with kinesin-AMPPNP complexes was first cross-linked with glutaraldehyde through Schiff base and Michael-type reactions,^24^ then immobilized onto 3-aminopropyltriethoxysilane (APTES) self-assembled monolayer (SAM)-derived surfaces and subsequently scanned (Fig. 1b and c respectively).

The representative morphology of a kinesin-inked microtubule pen is shown in Fig. 1d. Single kinesin molecules are seen as bead-like geometries distributed onto the linear structure of the immobilized microtubule; the control microtubule revealed a smooth, linear surface (Fig. 1d). Analysis also showed that such a bead distribution led to a local increase in the microtubule's horizontal diameter (Fig. 1e and f) from an average of 7.8 to an average of 14.4 nm (73 cases analyzed; herein the horizontal diameter is a reflection of the height changes as recorded by contact mode AFM). With such an increase, individual kinesin diameters varied between 2 and 8 nm. The observed variation in the kinesin diameter was presumably due to the different orientations that a bound molecule could assume, *i.e.*, straight or stretched along the microtubule's protofilament conformation^25^ respectively. Our analyses are supported by previous reports that showed that kinesin dimers immobilized onto microtubules led to an average of 5 nm changes in their diameters,^17^ an overall volume print of 6 × 3.5 × 3 nm^3^ (ref. 26) with Hu *et al.*^27^ showing size variations of 2–7 nm associated with single kinesin molecule orientation. Moreover, our analysis is supported by Kacher *et al.* and Schaap *et al.*, who noted that AFM can resolve single kinesin motors, with kinesins being visible as blobs when using AFM.^17,18^

**Fig. 2:**
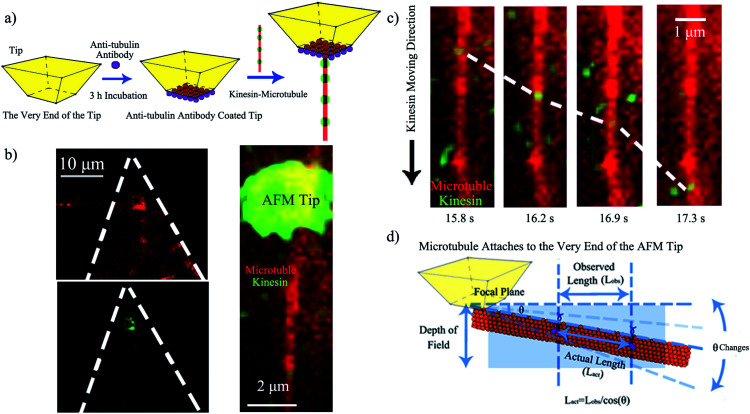
(a) Schematic of the AFM tip functionalization with anti-tubulin antibodies allowing controlled affixing of a kinesin-inked microtubule pen. (b) Left upper: optical microscopy image of a microtubule pen immobilized onto the AFM tip. Left lower: optical microscopy image showing green kinesin inks on the AFM affixed bio-pen. The white dashed lines depict the tip's profile. Right: representative fluorescence image of the inked microtubule pen affixed to the AFM tip. For this, merged images of red (microtubule) and green (labeled kinesin) were used. (c) Representative fluorescence images of kinesin (green) moving onto the affixed microtubule pen (red). The distance traveled by kinesin allows for recording of a measurable speed of about 3 μm s^−1^ (d) Relationship between the visually observed length and the actual length that kinesin could travel onto the affixed bio-pen. Different orientations of the affixed microtubule pen lead to different angles (*θ*) of observation.

Upon demonstrating kinesin's ability to recognize and ink a microtubule pen, we created a “holder” to affix such a bio-pen. The holder consisted of a biologically inert AFM tip functionalized with anti-tubulin antibodies (Fig. 2a), with the efficacy/reproducibility of the loading of the tip being controlled by the local distribution of the anti-tubulin antibodies. Fig. 2b shows the kinesin-inked microtubule pen affixed onto the AFM tip through site specific interactions based on ligand recognition reactions between tubulin (constituents of the microtubule pen) and anti-tubulin antibodies (immobilized at the tip) by optical microscopy. Nonspecific kinesin attachment (green tip co-localization) was noted, presumably due to kinesin's strong sub-micromolar binding affinity.^28^

Upon demonstrating the successful affixing of the kinesin-inked microtubule pen to the AFM tip, we evaluated the ability of the pen to “dispense” the ink. Herein dispensing is defined as the ability of kinesin to move along an affixed microtubule pen under the exchangeable ATP energy; it is known that kinesin takes single 8 nm steps with each such step correlated to an individual ATP hydrolysis event. Optical microscopy analysis confirmed the dispensing capability (Fig. 2c and ESI Fig. S1†). The average dispensing speed (defined as the total length that kinesin is able to travel onto an affixed microtubule pen over a user-recorded period of time) was 1.67 ± 1.42 μm s^−1^, as calculated from 29 independent measurements. The large variation in the dispensing speed was presumably due to the different orientations that an affixed microtubule could have (Fig. 2d) and/or the limited resolution available for single molecule optical signal processing. Specifically, an attached microtubule could show different orientation angles (*θ*) relative to the focal plane of the tip itself, with such angles leading to a variation in the observed length traveled (*L*_obs_) *versus* the actual length (*L*_act_) being recorded. The ability of standard microscopy to capture height distributions of specific fluorophore-labeled structures is known to be limited by the signal co-localization events, thus impeding the actual accurate observation.^29^

**Fig. 3:**
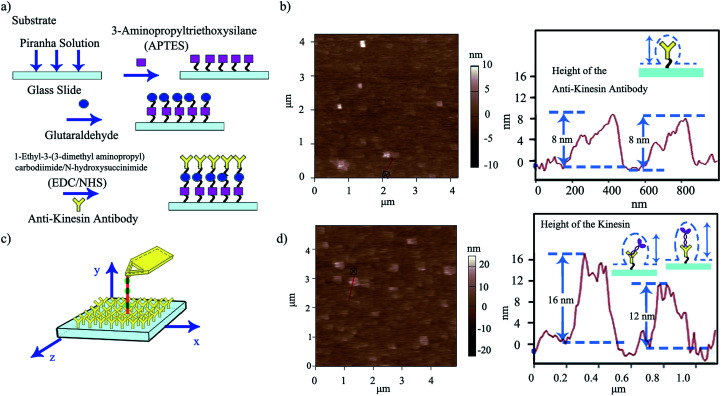
(a) Schematic of the multi-step functionalization of surfaces through zero-length chemistry and antibody binding APTES-based reactions. (b) Representative AFM morphology of anti-kinesin antibody coated surfaces and the resulting height distributions (depicted with the red line). (c) Schematic of single kinesin molecules written by the affixed microtubule pen. (d) Representative AFM morphology and distribution of single kinesin molecules written by the affixed microtubule pen and the height distribution of kinesin immobilized onto anti-kinesin antibody (depicted by the red line). Possible geometries and resulting anisotropies are discussed relative to the individual molecule packing.

Upon evaluating the ability of the affixed pen to dispense its ink, we developed a strategy for “capturing” the ink at specific locations on user-functionalized surfaces (ESI Scheme S1†). For this, we proposed that the affixed kinesin-inked microtubule pen (with kinesin bound in the AMPPNP state) would be brought in close proximity to an anti-kinesin antibody coated surface formed through a multi-step strategy using APTES as precursors. Aldehyde groups of glutaraldehyde were also used for zero-length chemistry and to allow the formation of imine linkages with the primary amine groups of the kinesins (Fig. 3a).

Anti-kinesin antibody surface functionalization was demonstrated using surface roughness analysis performed by contact mode AFM (Fig. 3b, ESI eqn (1) and (2)†), on (4 × 4) μm^2^ areas. Specifically, analysis of the surface height deviation (*i.e.*, *R*_q_)^30^ showed an increase in the surface roughness from 0.64 to 0.79 nm for the APTES-functionalized surface relative to the bare one (non-functionalized one; ESI Fig. S2a, b and S3a, b† respectively). Similarly, analysis of the arithmetic average of the absolute values of the surface height deviations measured from the mean plane (*i.e.*, *R*_a_)^30^ showed an increase in the surface roughness from 0.78 to 0.99 nm for the APTES functionalized surface again, relative to the bare one (non-functionalized one). *R*_q_ and *R*_a_ for the APTES-glutaraldehyde-anti-kinesin antibody functionalized surface were 1.12 and 1.54 nm (ESI Fig. S4a, b† respectively).

In contact mode AFM, the anti-kinesin antibody functionalized surface appeared generally smooth with isolated anisotropies of dots-like geometries (Fig. 3b). Surface analysis of such dots' geometries revealed heights between 2 and 8 nm (70 geometries evaluated). The recorded anisotropy is presumably due to binding occurring between the antibodies and APTES on the mica surface. Specifically, previous reports showed that APTES could assume five different orientations (*i.e.*, “single siloxane”, “double siloxane”, “triple siloxane”, “polymer” and “hydrogen bond” respectively, ESI Fig. S5†) at a glass slide interface,^31^ with such individual orientations impacting the subsequent height distribution of the molecule. Previous analysis showed that immobilized antibodies could also assume different orientations at a support interface from the “end-on fab-up”, to the “end-on fab-down”, “side-on” and/or “flat-on” geometry, with the individual molecular packing affecting individual antibody orientation and height as well as its individual rate and ability to bind other ligands.^32^

The dispensing and capturing, a.k.a kinesin writing, was subsequently proposed as being the stepping of a kinesin molecule off the inked microtubule when AMPPNP was exchanged with ATP, with such released kinesin to be captured by the proximal anti-kinesin antibodies (Fig. 3c and ESI Scheme S1†).^33^ Previous research showed that the stepping length of a single kinesin molecule is influenced by experimental conditions such as the ATP concentration, salinity and load sizes, just to name a few. In particular, Thorn *et al.*^34^ showed that single wild type kinesin had a stepping length of 1.0 ± 0.2 μm and 0.7 ± 0.1 μm, while single H1Q mutant kinesin had a stepping length of 5.8 ± 1.6 μm and 1.5 ± 0.5 μm before and after treatment with subtilisin respectively. Furthermore, Nishiyama *et al.*^35^ showed that the stepping length and velocity of kinesin are a function of its individual load. The authors estimated that a single kinesin at zero load had a run length of about 3.6 μm, at a velocity of about 930 nm s^−1^. The run length and velocity decreased to 0.2 μm and 230 nm s^−1^ for a 3.8 pN load and to zero when the load increased to 7.6 pN. Finally, intramolecular tension generated by the “neck linkers” was shown to affect the kinesin's stepping length.^36^ For instance, Yildiz *et al.*^36^ presented a wild type kinesin that had a run length of about 2 μm while the run length varied from about 1.5 to about 2.3 μm for a kinesin with different neck linkers.

Considering that the orientation of both APTES and anti-kinesin antibodies leads to anisotropic geometries,^37^ we further hypothesized that any kinesin dispensed from the microtubule pen as a pattern to be written onto the anti-kinesin antibodies would not only show an additional increase in local surface roughness at its place of binding, but also a surface dot-like geometry. Our hypothesis is supported by previous reports that showed that single molecules could be imaged using AFM,^17–21^ with kinesin molecules in particular being resolved by AFM as individual visible blobs.^17^

Indeed, kinesin writing led to an additional increase in surface roughness, *i.e.*, from 1.12 to 4.00 nm for *R*_q_ and from 1.54 to 5.02 nm for *R*_a_ for the APTES-glutaraldehyde-anti-kinesin antibody-kinesin surface relative to the APTES-glutaraldehyde-anti-kinesin antibody functionalized surface respectively (Fig. 3d, ESI Fig. S6 a, b†). The kinesin written onto the anti-kinesin antibodies assumed a dot-like conformation leading to recorded heights (horizontal diameter) between 4 and 17 nm (109 molecules evaluated; Fig. 3d). Such results are further supported by previous analysis that showed that immobilized kinesin could assume heights of 10.0 ± 1.8 nm for long or 8.6 ± 2.1 nm for short diameters.^38^ Others have also showed distributions of kinesins with a diameter of 9–10 nm through electron microscopy,^39^ with AFM studies supporting not only single kinesin measurements as listed in this analysis, but further single kinesin molecule dot-like shape.^17,18^

**Fig. 4:**
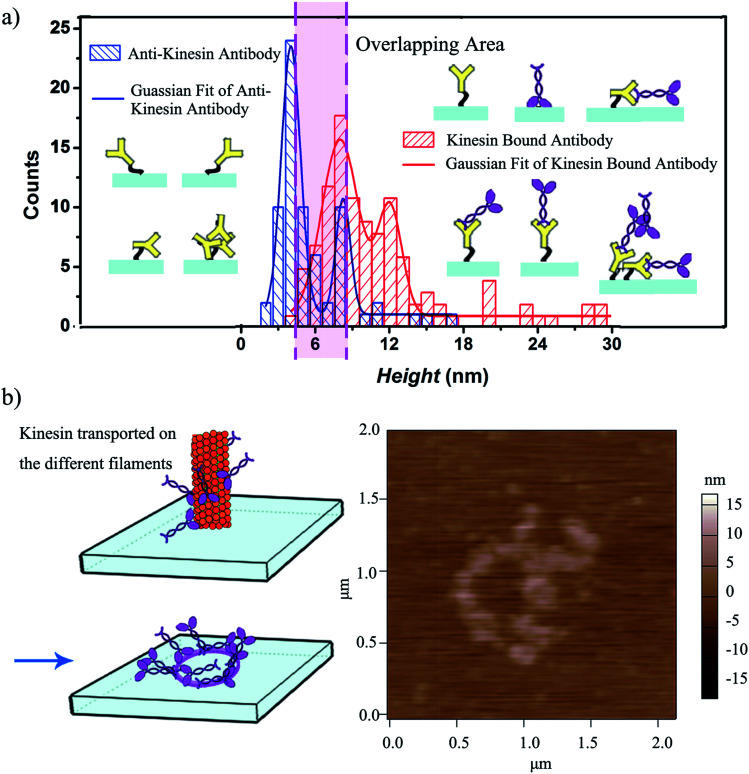
(a) Statistical variation of the height distribution of kinesin written onto anti-kinesin antibody and controls; 69 anti-kinesin antibodies and 104 individual kinesins written onto anti-kinesin antibody were evaluated. The possible geometries and resulting anisotropies are discussed relative to the individual molecule packing on the user-functionalized surface. (b) Kinesin molecules walk on different protofilaments of an affixed microtubule pen and are written onto anti-kinesin antibody functionalized surfaces (left) leading to circular pattern formation as identified by AFM (right).

The height variation observed for the written kinesin, similarly to the height variation of the antibodies/APTES surfaces, could be due to the packing of the individual molecule. Specifically, since kinesin could assume either a stand up or lay on geometry,^40^ the resulting kinesin–antibody conjugates could also assume geometries that mimic the "underneath" surface. Specifically, Fig. 4a shows the multi-Gaussian fittings of the height distributions for the two functionalized surfaces, namely anti-kinesin antibodies and kinesin-anti-kinesin antibodies. For the anti-kinesin antibodies, peaks heights varied from 2 to 17 nm with most heights smaller than 11 nm. The blue solid line resulting from the Gaussian fit identified the average height of the anti-kinesin antibody as being 5.54 ± 2.91 nm as well as two peak distributions at 4 and 8 nm respectively. These could be associated with a single anti-kinesin antibody with different orientations and/or anti-kinesin antibody conjugates respectively. For the kinesin-anti-kinesin antibodies, the heights changed from 4 to 29 nm, with a dominant regime in the 4–20 nm range. The red solid line showed the Gaussian fit results and identified an average height of kinesin-anti-kinesin antibodies of 11.26 ± 5.73 nm as well as two peak distributions at 8 and 12 nm respectively. Such peaks may be associated with the height of single kinesin-anti-kinesin antibody pair and/or kinesin-anti-kinesin antibody conjugates respectively. In addition, there was an overlap area (between 6 and 8 nm) presumably due to anti-kinesin antibody conjugates or single anti-kinesin antibody with a stand up orientation (*i.e.*, both “end-on fab-up” and “end-on fab-down” orientations). The heights of the kinesin-anti-kinesin antibodies could be associated with single kinesin-anti-kinesin antibody pairs with a lay orientation (kinesin bound with an antibody in “side-on” or “flat-on” orientation, single anti-kinesin antibodies and kinesin with a stand orientation, both “side-on” and “flat-on” orientations).

We further hypothesized that a longer contact time between the affixed bio-pen and the anti-kinesin functionalized surface will lead to writing capabilities that mimic the geometry of the kinesin distribution onto the bio-pen. Our analysis showed that allocating more time for writing indeed leads to controllable geometries of kinesin molecules on the user-functionalized surfaces. Specifically, upon increasing the writing time (*i.e.*, the time allocated for the affixed pen to be in close proximity of the anti-kinesin antibody coated surfaces), circular patterns of kinesin were obtained (Fig. 4b). Such geometries were presumably formed by kinesin molecule walking, dissociating and binding onto the anti-kinesin antibody surfaces from the different profilaments of the affixed microtubule pen. Such an assumption is based on previous research that showed that single kinesin molecules walk processively on one individual microtubule protofilament towards its positive end with such an analysis being supported through AFM studies.^17,18^ This assumption is also supported by our own analysis of the changes in the surface distribution with such changes being due to the longer time being considered for writing. Specifically, the representative height variations observed for the written kinesin in the circular patterns were about 9 and 15 nm respectively. The *R*_q_ and *R*_a_ were 4.38 ± 0.84 and 2.92 ± 0.76 nm for the area with the circle pattern and 1.92 ± 0.34 and 1.52 ± 0.13 nm for the area without the pattern.

The strategy presented herein is the first demonstration of a functional bio-pen capable of writing single molecules on user-designed surfaces. Such a pen could be used in the future to write both soft molecules and modified inks. For instance, one could envision functionalizing kinesin with nanospheres^41^ or CdSe quantum dot nanocomposites^42^ for parallel yet independent pattern formation, all with sub-nm resolution as indicated by the 8 nm step that a single kinesin could take. Moreover, one could envision controlling the ability to dispense such inks by controlling the chemical energy (a.k.a. ATP) provided at a given time in the writing process. Indeed, previous analysis^43^ showed that controlling ATP concentrations leads to differences in kinesin's speed (from 10 nm s^−1^ for 1 μM to 550 nm s^−1^ for 1000 μM of ATP being used). Furthermore, by controlling the pen's affixing, one could possibly control its dispensing ability/efficiency. For instance, using anti-gamma tubulin antibodies would ensure only immobilization of the minus end of the microtubule^44^ with its plus end being always exposed in close proximity of the user-functionalized surface to allow continuous writing of inked kinesins.^44^ By using contact mode AFM association and dissociation events, one could possibly evaluate and differentiate multi or single bond formation and their energy landscapes and how these depend on the specificity of the individual ligand–recognition reaction.^32^ Finally, it is envisioned that such a bio-pen could potentially have the ability for parallelization while providing individual writing ability with nanometer characteristics. Specifically, considering that a microtubule is formed from 13 independent protofilaments with each one of the possible filaments to be used by multiple inks^45^ at a given time and for multiple inking cycles, one could foresee the ability for controlled nanomanufacturing of such multitude of inks, all with ultrahigh resolution and for applications in nanoelectronics, pick and place nanorobots and even synthetic biology.

## Conclusion

Our study successfully produced a bio-pen using an AFM inert tip and molecular structures isolated from the cell. Our results showed that the ink (kinesin molecules) could be immobilized onto the bio-pen (microtubule affixed to the AFM tip) and autonomously written onto a user-functionalized anti-kinesin antibody coated substrate, all under the biochemical energy of ATP hydrolysis. We showed for the first time that the obtained biological pen has the ability to create different nanostructures with ultrahigh resolution on a user-functionalized substrate; these could to be used in the future in applications ranging from biosensing and high-throughput screening to nanomanipulation and structure formation.

## Conflicts of interest

There are no conflicts to declare.

## Supplementary Material

NA-002-C9NA00379G-s001
