# Supplementary material for: A bio-pen for direct writing of single molecules on user-functionalized surfaces
Source: Nanoscale Adv. 2019 Oct 31;2(1):156–65. doi: 10.1039/c9na00379g (PMC9417116; doi:10.1039/c9na00379g)
Supplement: NA-002-C9NA00379G-s001 [file NA-002-C9NA00379G-s001.pdf]

Electronic Supplementary Material (ESI) for Nanoscale Advances.
This journal is © The Royal Society of Chemistry 2019

Supporting Information

Bio-Pen For Direct Writing of Single Molecules on User-functionalized Surfaces

Xiao Hu[1] and Cerasela Zoica Dinu[1]*

[1]*Department of Chemical and Biomedical Engineering, West Virginia University, WV, USA, 26506*

***Corresponding author:**
Cerasela Zoica Dinu, Ph.D.
Department of Chemical and Biomedical Engineering
West Virginia University
Benjamin M. Statler College of Engineering and Mineral Resources
PO Box 6102
Morgantown, WV, 26506, USA
E-mail: cerasela-zoica.dinu@mail.wvu.edu
Tel.: +1 304 293 9338
Fax: +1 304 293 4139

1. Strategy for printing single kinesin molecules on user-functionalized surfaces

Inking of microtubule with kinesin molecules to form a bio-pen. The bio-pen is affixed onto an atomic force microscopy (AFM) tip to allow for regular geometries/ patterns of kinesin to be written on anti-kinesin antibodies user-functionalized substrates.

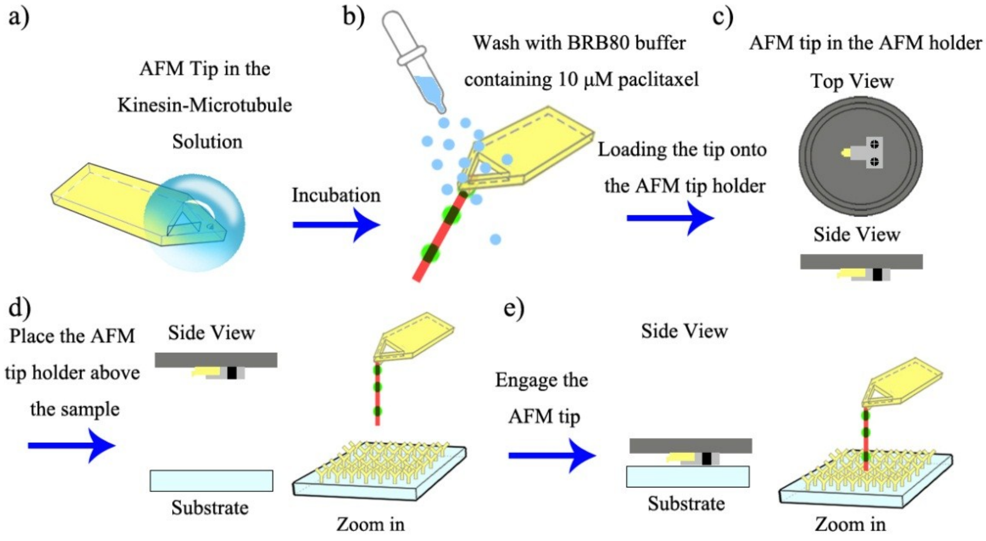

Scheme S1: Inking and AFM affixing of the bio-pen. Upon affixing, the tip is brought in close proximity to anti-kinesin functionalized surfaces to allow single kinesin molecule printing.

2. Length of the microtubule

Microtubule length was evaluated using optical microscopy. For this, microtubules were first synthesized from free tubulin suspended in a microtubule polymerization solution according to established protocol[3]. Briefly, the polymerization solution was obtained by vortexing 5 µL 100 mM $MgCl_2$, with 6 µL dimethyl sulfoxide (DMSO, 99.7%, Fisher Scientific, USA), 5 µL 25 mM guanosine-5'-triphosphate (GTP, Sigma, USA) and 9 µL BRB80 buffer (formed from a mixture of 80 mM piperazine-N,N'-bis(2-ethanesulfonic acid buffer, 1 mM $MgCl_2$ and 1 mM ethylene glycol tetraacetic acid (EGTA), pH 6.8; all reagents were purchased from Fisher Scientific, USA). To initiate microtubule polymerization, 2.5 µL polymerization solution was mixed with 10 µL of 4 mg/mL biotin and rhodamine labeled tubulin (Cytoskeleton Inc, USA) and the mixture was incubated at 37 ^{o}C for 30 min. To stabilize the resulting microtubules, the solution was dispersed in 1 mL BRB80 buffer containing 10 µM paclitaxel (Fisher Scientific, USA). The stabilized rhodamine labeled microtubules were analyzed under a 100 x objective (NA=1.4) on an MFP-3D Bio containing an integrated Nikon (Asylum/ Nikon, USA) and under the DAPI filter and an exposure time of 8.3 s. Average length distributions were 18.94 nm±8.41 nm (210 microtubule analyzed).

3. Kinesin *in vitro* stepping assay

Kinesins' ability to bind and step off the lab-synthesized microtubules was evaluated. First, kinesin binding to the microtubules was evaluated by mixing 8 µL of 10 µg/mL of kinesin expressed in the lab (see Materials and Methods section of the manuscript) with 2 µL 20 mM adenylyl-imidodiphosphate (AMP-PNP, Sigma, USA) and incubating the mixture for 1 h at 4 ^{o}C. The mixture was subsequently mixed with 20 µL microtubules (prepared as described in the manuscript in the Materials and Methods section) and 10 µL of BRB80 buffer containing 10 µM paclitaxel; resulting solution was incubated for 30 min at room temperature. Upon incubation, the unbound kinesin was separated in the supernatant by using an Allegra 64R centrifuge (Beckman Coulter, USA) and 30,000 rpm spinning for 10 min.

Secondly, kinesin stepping off the microtubule was evaluated upon suspending the inked microtubule (i.e., inked bio-pen) as indicated in the Schematic 1 above and using an ATP-based assay[4]. For this, 100 µL gliding solution (BRB80 buffer containing 1mM MgATP, 20 mM D-glucose; both reagents were from Fisher Scientific, USA), 0.02 mg/mL glucose oxidase, 0.8 mg/mL catalase (both reagents from Sigma, USA) and 0.5% β-mercaptoethanol (Fisher Scientific, USA) were added onto the tip functionalized with the kinesin-inked microtubule and incubated for 5 min at room temperature. The writing process was observed using a fluorescent microscope and a 100 x objective (NA=1.4) under the DAPI and GFP filters and under an exposure time of 8.3 s. Other representative images of stepping assay are included below.

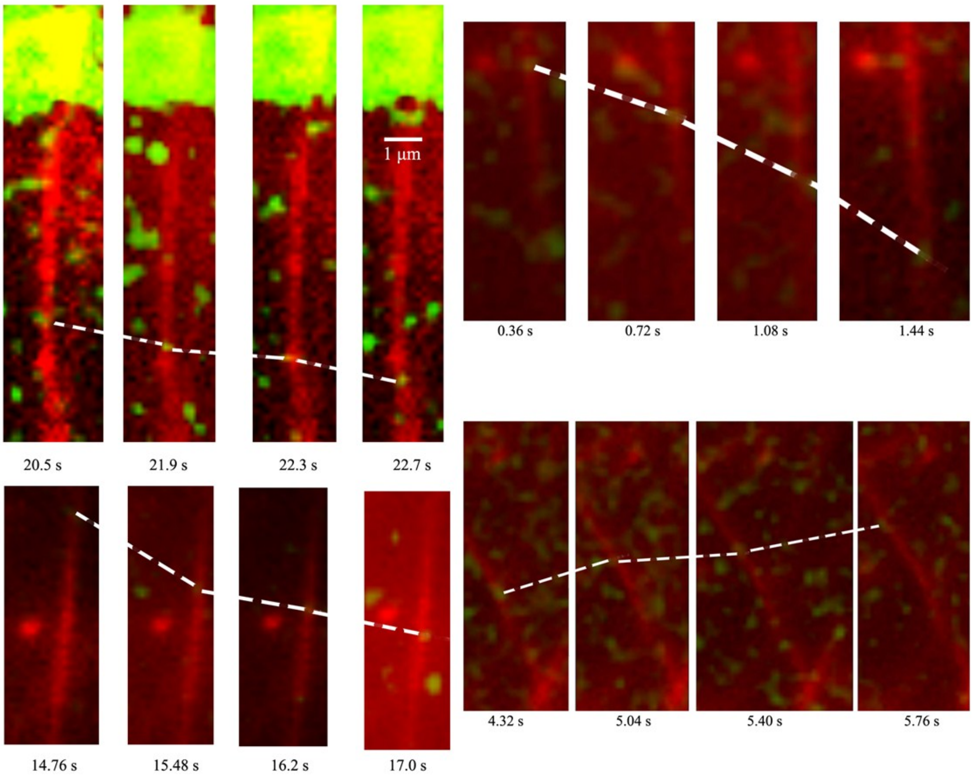

Figure S1: Time frame fluorescence images of the stepping assay from parallel/ individual experiments. Kinesin (green labeled) is moving on immobilized microtubule (red).

4. Roughness factors (R_q and R_a)

Roughness factors R_q and R_a defined as the root mean square average of height deviation (as taken from the mean image data plane) and the arithmetic average of absolute values of the surface height deviations (as measured from the mean plane) respectively, and were calculated using equations 1 and 2 below:

$$R_q = \sqrt{\frac{1}{l}\int_0^l Z^2(x)dx} \quad \text{(Eq.1)},$$

$$R_a = \frac{1}{l}\int_0^l |Z(x)|dx \quad \text{(Eq.2)}$$

Herein l is the total sample length and Z is the sample height[1].

5. Atomic Force Microscopy (AFM) morphology analyses

5.1. Clean glass substrate

The morphology of a glass slide was evaluated using contact mode AFM and an MFP-3D Bio (Asylum, USA). For this, the glass slide (d=25 mm, Corning, USA) was first ultrasonicated in DI water, 99% ethanol (90%, Fisher Scientific, USA), and again in DI water, with 30 min for each of the sonication window. Secondly, the slide was dried under vacuum for 1 day and subsequently exposed to UV light for 30 min. The morphology of the glass slide was evaluated by contact mode AFM in liquid. For such analyses, the cantilever's spring constant was calibrated using the thermal noise method; the scan rate of the tip was fixed at 0.5 Hz for all the experiments. At least 6 samples have been investigated to collect individual morphologies and results are shown in Figure S2. R_q and R_a of the clean glass substrate were calculated as listed above to result in 0.64 and 0.78 nm respectively.

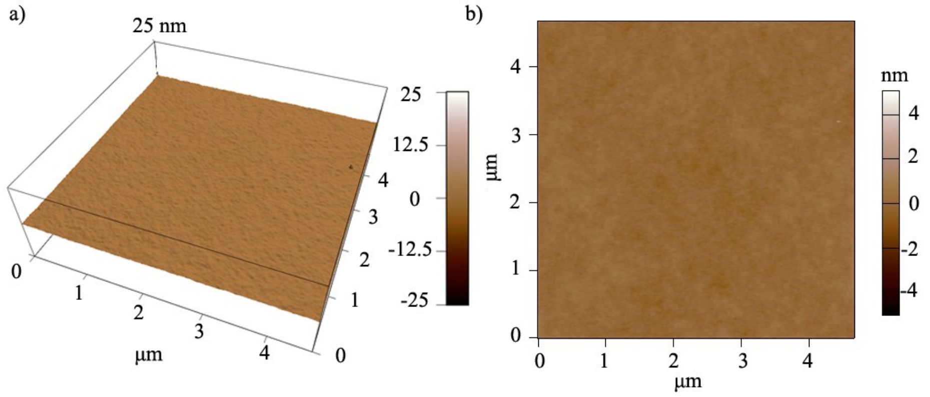

Figure S2: (a) Representative 3D AFM morphology analysis of a clean glass substrate. (b) Representative 2D AFM morphology analysis of a clean glass substrate.

5.2. Functionalized glass surface

The morphology of the 3-aminopropyltriethoxysilane (APTES) functionalized surface was also evaluated according to the protocol described above. Herein the APTES functionalization was obtained by immersing the clean glass in 1 ml of 5% APTES in toluene (99.5%) with incubation at room temperature for 1 h. Upon time elapsed, the slide was

washed thoroughly with DI water, toluene and again DI water to remove any unbound APTES. R_q and R_a of the APTES functionalized substrate were calculated from the Figure S3 and equaled to 0.79 and 0.99 nm respectively.

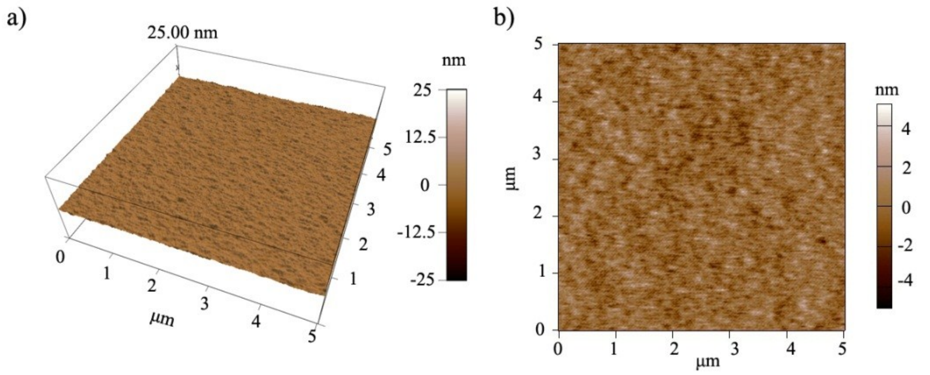

Figure S3: (a) Representative 3D AFM morphology of the APTES functionalized substrate surface. (b) Representative 2D AFM morphology of the APTES functionalized substrate surface.

5.3. APTES-glutaraldehyde-anti-kinesin antibodies functionalized surface

The morphology of the APTES-glutaraldehyde-anti-kinesin antibodies functionalized surface was also evaluated according to the protocol described above. Herein the glutaraldehyde-anti-kinesin antibodies functionalization was obtained upon immersing the APTES functionalized glass slide into 1 mL 5% glutaraldehyde in 0.2 M pH 9.0 Tris-buffered saline (TBS) at room temperature, for 1 h, with shaking at 200 rpm. Subsequently the slide was extensively rinsed with TBS, activated for 15 min in a 1 mL 160 mM 1-Ethyl-3-(3-dimethylaminopropyl)carbodiimide and 80 mM N-Hydroxysuccinimide (NHS) in 2-(N-morpholino)ethanesulfonic acid buffer (MES, pH 4.7,) and incubated in 1 mL of 1 mg/mL anti-kinesin antibody in BRB80 at room temperature and 200 rpm for 3 h respectively. Upon incubation, the functionalized glass slide was rinsed thoroughly with BRB80 to remove loosely bound antibodies. R_q and R_a of the APTES-glutaraldehyde-anti-kinesin antibodies functionalized substrate were calculated and equaled values of 1.12 and 1.54 nm respectively.

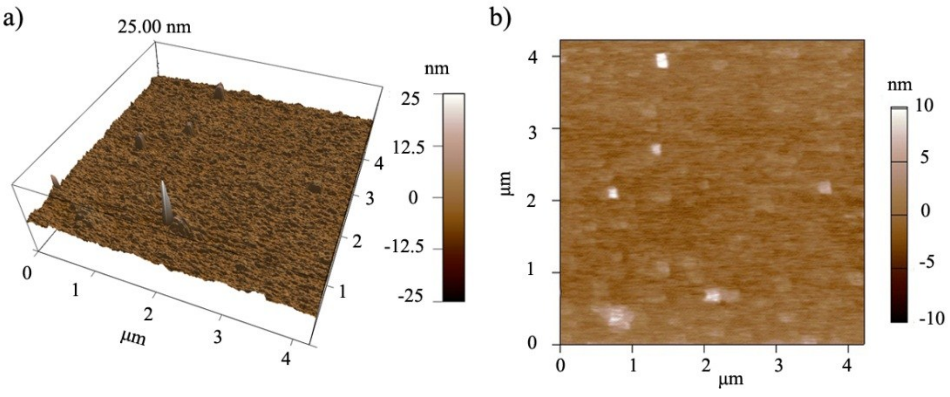

Figure S4: (a) Representative 3D morphology of APTES-glutaraldehyde-anti-kinesin antibodies functionalized surface. (b) Representative 2D morphology of the APTES-glutaraldehyde-anti-kinesin antibodies functionalized surface.

6. Possible orientations of the APTES on the glass slide

According to previous research[2], APTES molecules could potentially assume five different orientations upon immobilization onto glass slides. These are herein defined as "single siloxane", "double siloxane", "triple siloxane", "polymer" and "hydrogen bond", as resulting from the groups presumed to drive such interactions. Each orientation shows different height that could lead to the observed height variation recorded using AFM analysis (Figure S5).

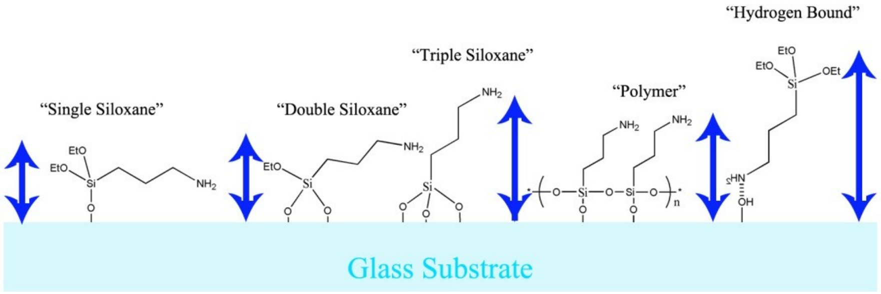

Figure S5: Possible orientations of the APTES at the glass substrate interface.

7. AFM morphology analyses of the APTES-glutaraldehyde-anti-kinesin antibodies-kinesin functionalized surface

The morphology of the APTES-glutaraldehyde-anti-kinesin antibodies functionalized surface was evaluated according to the protocol described above. Herein the kinesin had to be printed using the protocols enlisted in the main paper. R_q and R_a of the APTES-glutaraldehyde-anti-kinesin antibodies-kinesin functionalized substrate were calculated and equaled 4.00 and 5.02 nm respectively (Figure S6).

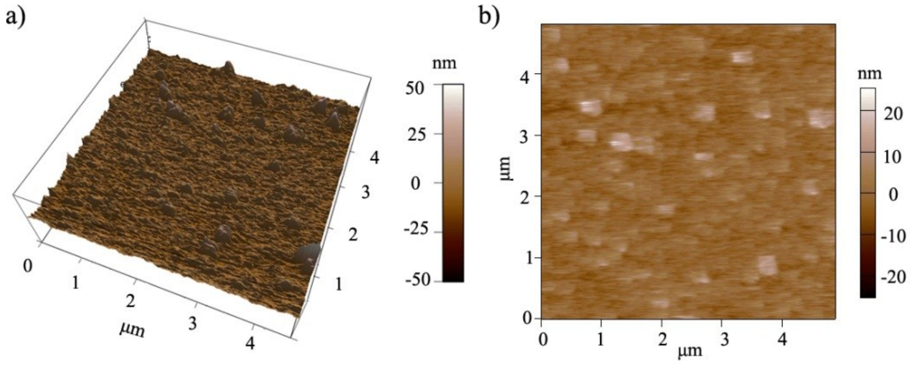

Figure S6: (a) Representative 3D AFM morphology of the APTES-glutaraldehyde-anti-kinesin antibodies-kinesin functionalized surface. (b) Representative 2D AFM morphology of the APTES-glutaraldehyde-anti-kinesin antibodies-kinesin functionalized surface.

References:

1 Gadelmawla, E. S., Koura, M. M., Maksoud, T. M. A., Elewa, I. M. & Soliman, H. H. Roughness parameters. *Journal of Materials Processing Technology* **123**, 133-145, doi:https://doi.org/10.1016/S0924-0136(02)00060-2 (2002).

2 Acres, R. G. *et al.* Molecular structure of 3-aminopropyltriethoxysilane layers formed on silanol-terminated silicon surfaces. *The Journal of Physical Chemistry C* **116**, 6289-6297 (2012).

3 Hu, X., Dong, C., Su, R., Xu, Q. & Dinu, C. Z. Protein self-assembly onto nanodots leads to formation of conductive bio-based hybrids. *Scientific reports* **6**, 38252 (2016).

4 Dinu, C. Z. *et al.* Parallel Manipulation of Bifunctional DNA Molecules on Structured Surfaces Using Kinesin-Driven Microtubules. *Small* **2**, 1090-1098 (2006).
